# The Gloucestershire Royal Infirmary and Eye Institution

**Published:** 1915-05-22

**Authors:** 


### THE DEPARTMENT OF MODERN NURSING.*

### XIII.—The Gloucestershire Royal Infirmary and Eye Institution.

For this hospital, which uses on an average a hundred out of its full number of 140 beds, there is a nursing staff of thirty-three, composed of four sisters, two staff nurses for the children's ward and the theatre, twenty-seven probationers, and a night sister. The sisters are sometimes, though not invariably, chosen from nurses in the institution after they have had an interval of other work.

The agreement that has to be copied out and signed by the probationer sets forth that she must remain in the service of the hospital for one year at the completion of the three years' training, either on hospital duty or on the private nursing staff. Until lately no salary was given for the first year, but in consequence of a great difficulty in finding suitable candidates the sum of £4 is allowed for the first year, rising in the following years to £8, £12, and £24. The same dearth of candidates renders it impossible to insist on any fixed standard of education, and although there is a printed limit of age to the years between twenty-two and thirty, this is not rigidly insisted on.

The surgical training to be obtained here is particularly good, partly on account of the number of serious mining and pit accidents received from the Forest of Dean. All the probationers take theatre duty in the course of their training, mostly in the latter part of it, and a probationer goes to the theatre with every operation case from her ward. Much eye work is done in the hospital, and most of the nurses have the opportunity of seeing it. There is also an *x*-ray department, but in this treatment the probationers have only the part of onlookers should they accompany patients from the wards. A probationer is on duty in the out-patient department, and all have a turn in the children's ward. As is usual in country hospitals, they also get a good deal of experience in the preparation of patients for operation and in wound dressing, urine testing, etc.

Each candidate is received for a trial month, which period is usually sufficient to weed out the undesirable and unwilling. Most of those who survive the month stay to finish four years' work. The candidate pays 12s. 6d. to the hospital for materials for uniform to be made by herself for wear during the month. If she stays on, the money is repaid to her and uniform is found for the rest of the time. At the end of the month the candidate is interviewed by the committee.

Lectures are given by the assistant matron during the first year on elementary nursing and bandaging, and by the matron on more advanced nursing in the second year. The house surgeon and house physician give lectures on anatomy and physiology, and the theatre nurse holds classes on instruments. At the end of the second year there are examinations on all these subjects, oral, written, and practical, which every nurse is required to pass before receiving her certificate. Massage is not taught. The probationers attend lectures on sick cookery at the City School of Cookery. Much pains are bestowed on the classes held after the lectures given by the resident staff. Notes of the lectures are thoroughly worked up, and difficulties are explained until even the densest brain has had the subject made clear.

The nurses live in a comfortable home at one side of the hospital grounds, with the advantage of an enclosed garden where they can take off their caps, rest in a summer house, and play tennis if so inclined. Their meals are taken in the hospital, where also for want of sufficient room some of the juniors are housed in the upper storey. The assistant matron looks after the home and its inmates. There is a private staff in connection with the hospital, who also live in the home and have a sitting-room to themselves.

Two hours off duty are allowed daily to the probationers, and three hours on Sundays. One whole day a month is also allowed, and it is not spoiled, as is too often the case, by a short spell of work in the morning. A fortnight's holiday is given in the first and second years, and three weeks in the third.

Nothing is done at present in the way of helping the staff to join the pension fund. The question has been discussed, and it is not unlikely that something may be arranged, but in any case it will not be compulsory. It is found that with the small salary that the nurses receive for the three years they cannot spare anything beyond the necessary 3s. 3d. quarterly for insurance. It appears to us that this is a good reason for giving substantial aid in the payment of premiums.

* Previous articles: May 16, 30; June 13; July 11, 25; Aug. 8, 1914; Jan. 16, 30; Feb. 27; Mar. 13; April 3, May 8, 1915.

